# Applicability of working abroad for physicians with a specialization in Global Health and Tropical Medicine

**DOI:** 10.1186/s12992-023-00929-6

**Published:** 2023-04-20

**Authors:** Hasan Özcan, Loes Overeem, Maria Bakker, Caroline Telkamp, Robbert Duvivier, Janine de Zeeuw, Marco Versluis

**Affiliations:** 1 Radboud University, Nijmegen, The Netherlands; 2 University of Groningen, Groningen, The Netherlands; 3 Department of Paediatrics, Maasstad Ziekenhuis, Rotterdam, The Netherlands; 4 Department of Obstetrics and Gynaecology, St. Walburg’s Hospital, Nyangao, Tanzania; 5 Department of Health Sciences, University Medical Center Groningen, Groningen, The Netherlands; 6 Department of Obstetrics and Gynaecology, University Medical Center Groningen, Hanzeplein 1, Groningen, 9713 GZ the Netherlands; 7 Department of Health Sciences, Global Health Unit, University Medical Center Groningen, Groningen, The Netherlands

**Keywords:** Value of working abroad, Professional development, Cultural development, Personal development, Competency development, P-GHTM

## Abstract

**Background:**

In The Netherlands, physicians specialized in global health and tropical medicine (Ps-GHTM) are trained to work in low-resource settings (LRS) after their training program of 27 months. After working for a period of time in LRS, many Ps-GHTM continue their careers in the Dutch healthcare system. While there is limited evidence regarding the value of international health experience for medical students and residents, it is unknown to what extent this applies to Ps-GHTM and to their clinical practice in the Netherlands.

**Methods:**

In this qualitative study we conducted semi-structured interviews and focus group discussions (FGDs) with Ps-GHTM to explore the perceived applicability of their experience abroad for their subsequent return to the Netherlands. Topic guides were developed using literature about the applicability of working abroad. Findings from the interviews served as a starting point for FGDs. The interviews and FGDs were analysed using directed content analysis.

**Results:**

15 themes are described relating experience abroad to healthcare delivery in The Netherlands: broad medical perspective, holistic perspective, adaptive communication skills, creativity, flexibility, cultural awareness, self-reliance, clinical competence, cost awareness, public health, leadership, open-mindedness, organization of care, self-development, and teamwork. Highlighting the variety in competencies and the complexity of the topic, not all themes were recognized by all respondents in the FGDs nor deemed equally relevant. Flexibility, cultural awareness and holistic perspective are examples of important benefits to work experience in LRS.

**Conclusion:**

Ps-GHTM bring their competencies to LRS and return to the Netherlands with additionally developed skills and knowledge. These may contribute to healthcare delivery in the Netherlands. This reciprocal value is an important factor for the sustainable development of global health. Identifying the competencies derived from work experience in LRS could give stakeholders insight into the added value of Ps-GHTM and partly help in refining the specialization program.

## Introduction

In an increasingly interconnected and interdependent world, global health (GH) [1] is becoming ever more important [1–4]. The COVID-19 pandemic is an example in which health issues transcend beyond national borders. Aside from the pandemic, GH also encompasses different infectious diseases, trends in non-communicable diseases, cross-cultural interactions and various healthcare systems and requires physicians having the understanding of working in a global environment [2, 3, 5, 6].

The rise of GH historically has a strong connection with colonialist attitudes [7, 8]. Understanding the role of colonialism is important to address health inequity and the calls for decolonizing GH have been growing recently to move towards health equity and sustainable partnerships with mutual benefits [9–16]. It is clear that medical tourism can be harmful, especially when performed by unsupervised students [17, 18] or even laypersons [19]. In addition, physicians working in these settings, even with good intentions, may also have a negative impact [20, 21].

Although training experiences in GH can offer benefits to both sending and receiving party, these experience may also raise ethical challenges. These challenges include burdens on hosts in LRS, concerns regarding sustainability, and negative impact on patients, communities and local trainees [18, 22]. Therefore, there is an increasing demand that clinical work abroad should be guided by the GH ethics and aligned with the needs and priorities of local healthcare systems [18, 22–24]. This is a complex ethical discussion that requires an understanding of the needs and priorities of receiving parties as well as the implications for sending parties in order to come to a sustainable collaboration [14, 17, 25].

In the Netherlands, medical doctors can specialize as a physician in Global Health and Tropical Medicine (P-GHTM). This post-graduate training programme consists of training in obstetrics and surgery or paediatrics in Dutch hospitals, followed by a GH curriculum of 3 months and concluded with a period of training abroad under supervision. After training for 27 months the Ps-GHTM are equipped to contribute to healthcare delivery and capacity building as well as the ethical aspects of their work in low-resource settings (LRS) [26]. The training institute in Global Health and Tropical Medicine (OIGT) directs the training of Ps-GHTM and is currently investigating the needs and priorities of local partners in order to improve training of Ps-GHTM. After their training, most Ps-GHTM work abroad for several years before returning to the Netherlands to continue their career. The experience gained abroad can be of benefit to health care in high-resource settings (HRS). Understanding the needs and priorities of local partners, as well as the benefits of experience abroad can facilitate an open discussion on reciprocal collaboration. However, it is unclear what competencies are developed abroad and how they may contribute to healthcare in HRS.

We did not find prior studies investigating competency development by Ps-GHTM, or other physicians in GH, during time spent working in LRS. However, there are studies describing how experiences during international health electives (IHEs) contribute to competency development for students and medical residents [2, 27–34]. Roy et al. describe three domains in which outcomes of international mobility programs for students can be categorized: personal, professional, and cultural [32]. Studies on IHEs have found that students had increased self-confidence, self-reliance, and self-efficacy, along with improved academic performance, enhanced physical examination skills, and increased affinity with under-served populations by experiences in LRS [2, 32]. For residents in general practice, enhancement of soft skills, overcoming language and cultural barriers, increased confidence in practicing medicine, increased perspective of healthcare systems, and improved management of (scarce) resources was reported [31]. In addition, improved resourcefulness and cost-effectiveness, improved medical knowledge, enhanced procedural skills, and better physical examinations was described for medical residents participating in such IHEs [29].

These studies suggest that the challenges posed by working in LRS, such as working with fewer resources, dealing with cultural differences and differences in disease presentation, facilitate competency development. Moreover, the importance of reciprocal value in collaboration between partners in high, middle and low income countries is increasingly recognized [6, 15]. As such, competencies developed during a period of work abroad, can contribute to professional performance when healthcare workers continue their career in HRS. Describing competency development can help clarify the benefits of experience abroad which in turn can facilitate sustainable partnerships. Many Ps-GHTM who were training or working in the Netherlands were called in to assist in Dutch hospitals during the COVID-19 pandemic based on their assumed competencies in crises, triage and healthcare management. This unique setting provided the opportunity in which the applicability of the gained competencies could be explored. This study aims to explore the competencies of Ps-GHTM gained during their work abroad in LRS and the perceived applicability of these competencies in the Netherlands.

## Methods

### Study design

This is a qualitative, explorative study with interviews and FGDs to provide a rich description of perspectives on the competencies gained by participants during their training and period abroad.

### Study population and sampling

Participants were Ps-GHTM who (i) enrolled in and graduated from post-graduate training from 2007 and onwards, (ii) who had worked abroad after graduating, and (iii) who worked (either in the Netherlands or abroad) during the COVID-19 pandemic.

Initially, 61 potentially suitable Ps-GHTM were approached in July 2020 through the Dutch Association of Tropical Medicine and Global Health (NVTG) and OIGT and asked if they were interested in participation. Ps-GHTM that responded positively received an information letter and informed consent. Further recruitment took place through snowball sampling using social media [35]. We aimed to include 20–30 Ps-GHTM as we expected to reach theoretical saturation with this sample size [36]. A total of 33 semi structured interviews and four FGDs were held between July until November 2020.

The study population includes 25 Ps-GHTM between 30 and 68 years old (median = 33, interquartile range (IQR) = 3,5 years.) who graduated between 2009 and 2020. The age of two participants was unknown. Ratio female-male: 17 − 8. Participants have worked in various LRS such as in India, Ghana, and Sierra Leone. The time spent working abroad at the time of the interviews ranged from 7 months to 7 years (mean = 15 months, IQR = 17 months). 24 participants worked in The Netherlands during the COVID-19 pandemic, 1 participant worked in the Gambia.

### Scoping search and development of topic guides

We conducted a scoping review on competency development during electives or work abroad. This was done in order gain a general understanding of the topic and to be able to develop topic guides for the semi-structured interviews and FGDs [36, 37]. According to the literature, healthcare workers are suggested to develop a variety of competencies in their time abroad. These include: improvement of cultural and interpersonal competency, professional and career development, improved understanding of healthcare systems as well as suggested competencies recognised within HRS such as leadership, collaboration and scholar as described in CanMEDS, improvement of knowledge and skills, improvement of resourcefulness and/or cost-effectiveness [29, 31, 38]. Based on this literature, a preliminary framework was developed through deductive category development. This allowed the researchers to focus on the research question and identify key concepts as initial coding categories, which were then categorized in three domains (cultural, personal, professional) as described by Roy et al [32, 39]. The topic guides were initially developed by LO and HO and were peer debriefed with the research team. Semi-structured interviews and FGDs were chosen, as these allow participants the freedom to explain their experiences in their own words, increasing the resolution of details provided [36]. Following a constructivist approach, participants’ perspective was investigated while gaining insight based on the experiences of these participants and the interaction between participants [40].

### Data collection

#### Semi-structured interviews

A pilot interview was conducted to reflect on the process of interviewing and to analyse the feasibility of the questions prior to the start of data collection. All consecutive interviews were set up online using the application ‘Zoom’, or if requested ‘Skype’. The interviews took between 45 and 90 minutes. The pilot interview was not analysed. The guide was not static and was periodically reflected upon based on active reflection by the research team [37, 41]. Periodical reflection allowed for new items that were not previously described, to be included in the interview.

#### Focus group discussions

FGDs were held to confirm the data, gain more in-depth understanding of the data, reflect on our interview guide as described previously, define competencies and their themes, and to explain causes and effects of these competencies and themes [36]. By analysing the different perspectives and the conversational exchanges of participants, we defined competencies and themes and explained their causes and effects. The discussions were structured around five selected themes in a FGD guide, based on the most recurring and prominent themes from the interviews. Initially, these themes were distilled from the first six interviews: leadership, communication, organisation of care, cultural awareness and flexibility. Open questions were followed by more theme-specific sub questions to crystallise elaborate information.

Participants of the FGDs were sent ground rules beforehand as the FGD took place via ‘Zoom’. The FGDs lasted between 90 and 120 minutes. All participants were asked to partake in the FGDs, but were selected based on their availability on the particular dates. The size of the FGDs were, in chronological order: 4, 6, 4, 6. A total of 13 interviewees did not participate in the FGDs.

The discussion guide was reflected upon before and after every new FGD, as an iterative process. Based on these reflections, changes as rephrasing questions and restructuring order of questions were made [37, 41].

### Data handling

All data was collected online through the internal recording software of video conference platforms. The audio-visual recordings were stored in a secured digital location in accordance with European General Data Protection Regulation. Transcripts were anonymised using a separate key document. For transcription purposes, files were sent through a password-protected portal. The datafiles (audio recordings, transcripts, and key-document) will be saved for ten years after which they will be deleted, as stipulated by Dutch privacy law. The participants of this study did not gain any direct benefits by participating in this study and received no compensation.

### Data analysis

In total, 25 interviews and four FGDs were analysed. Up to the 27th transcript, including 23 interviews and four FGDs, new codes were created. After analysing another two interviews without creation of new codes nor themes, we concluded to have adequate information power. As such, the remaining transcripts were not analysed.

Conducting interviews and FGDs as well as coding and checking were done by HO and LO. The transcripts were coded in Atlas.Ti and analysed using directed content analysis based on the preliminary framework that was established. This iterative process was guided by the following steps [37, 39, 41].

After six interviews, explorative coding was done to familiarize the researchers with the main topics discussed in the interviews. At this stage, both researchers coded all interviews separately and discussed their findings with the research team. In an iterative deductive process, transcripts were coded and discussed. After the tenth interview, both researchers coded different interviews individually and checked each other’s work. The codes were intermittently peer debriefed to the research group for further specification.

Main themes were created to categorise the codes and discussed within the team. The codes were periodically reflected upon and merged or split when deemed necessary. The first FGD started after six interviews, after which the interviews and FGDs were both conducted interchangeably.

The framework of themes and codes was first assessed using the next 10 interviews to test the validity and vice versa. Both researchers each coded half of the transcripts individually and checked each other’s work. The framework was specified through this process as some codes required change of themes or new themes to fit into the framework. The framework was peer debriefed regularly.

After this, HO coded the remaining transcripts until no new codes were created. At this point, the codes within each theme were expanded, but no new themes were found. The results were peer debriefed.

## Results

**Fig. 1:**
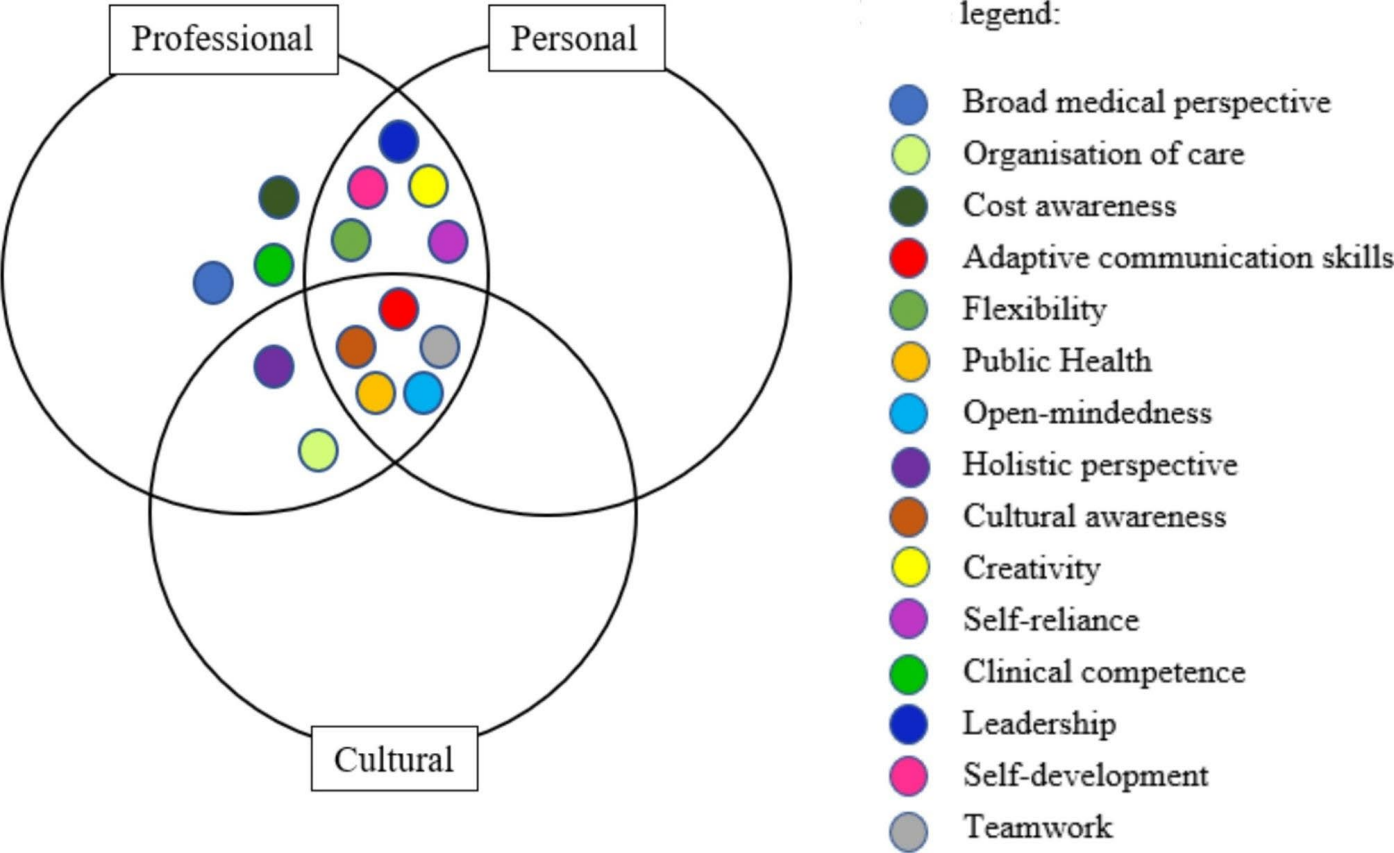
Venn diagram of all themes within their respective domains *Description: A visual overview of the themes within their corresponding domains.*

All participants perceived benefit from their experience abroad for their work in HRS. A total of 15 distinctive themes were identified after analysis of the transcripts. These themes describe the competency development during work abroad as perceived by Ps-GHTM. Below we describe five themes by using quotes from participants and describe corresponding competencies to illustrate how the competencies are derived. The quoted participants are referred to as: Px. The themes relate well to the domains of competency development as described by Roy et al., as illustrated on Fig. 1. Some themes related to more than one domain. For example, creativity can be of benefit to a P-GHTM in both professional and personal spheres. All themes are summarised in Table 1.

### Cultural awareness

Ps-GHTM are experienced in working in teams with people with different cultural backgrounds. Participants reported being able to adjust their approach of others (e.g., team/team members, patients, patient family) to local norms without imposing their own standards upon others, acknowledging that these could be improper in the new context. Ps-GHTM reported adopting a humble and respectful attitude towards the hosting culture, which also increased adherence of the patient to their medical advice.

“You find out that you have a different perspective on things and so are forced to reflect on why things are done in a specific way in your own culture and that other approaches may be possible” (P41).

### Adaptive communication skills

Being dependent on team members with different backgrounds and attitudes and having patients with different languages and cultures stimulated Ps-GHTM to reflect on their own ways of interaction and to expand their communication arsenal. Participants reported consciously adapting to the level of training of their team members to establish mutual understanding, even in acute situations. Interviewees emphasised the importance of communication in healthcare delivery, having experienced lack of proper communication, such as cultural and language barriers, as an obstacle for proper healthcare delivery.

“Working with so many different colleagues from different cultures speaking different languages, you develop a certain kind of flexibility in the communication, which could come in handy in the Netherlands with various colleagues but also with a variety of patient groups. You try to adapt to their level of understanding of a language and to their ideas” (P02).

### Open-mindedness

Having experienced various traditions and habits, Ps-GHTM report being more open and less judgemental to different views in life, such as alternative medicine. They also reported that, due to intense experiences like frequent deaths, being able to put matters into perspective and understand that the world is not defined by their own views.

“(…) taking into account the perceptions of people and being non-judgemental and curious about it. ‘What makes a person go to an alternative healer? What moves a person to do what they do?‘ Being more curious about it. That strengthens the communication, because people somehow feel that you don’t judge them.“ (P02).

### Public Health

Ps-GHTM worked in settings where providing adequate basic healthcare was difficult, which highlighted the importance of public health to them. Participants report an increased affinity with vulnerable populations and reported being motivated to provide suitable healthcare to them.

“’Wanting the perfect treatment for that one patient, whilst not having enough capacity to treat everyone. What is the best option for all? Who really needs treatment, who doesn’t?‘ I have more experience in that area.“ (P07).

“I want to try my best to serve vulnerable populations, like refugees or the homeless, either abroad or in a deprived neighbourhood. To somewhat keep the spirit of what they do in northeast India” (P02).

### Teamwork

Ps-GHTM describe valuing diversity within a team and being able to work in a diverse team, having a structured way of working and solving conflict. Moreover, they report considering the expectations of their team members to manage common goals. Ps-GHTM mentioned that they adjust their way of teamwork to the needs of the team, whether this is skills-, knowledge- or communication-wise.

“(…) I prioritise things and also communicate that to others, as in: ‘what do we want? What is the most important matter to solve? What are the most urgent issues?‘ I involve others in this. That’s where teamwork reappears, because you cannot do this alone.“ (P03).

**Table 1:** ; Theme descriptions, assigned domains and applicability. *Description: A list of all 15 themes, their definitions as derived from the data, the domains to which the themes are assigned to, and all competencies described as their potential applicability in the Netherlands*

	Theme	Definition	Domain	Reported applicability / Examples
1	Broad medical perspective	Thinking and acting beyond the silo of singular medical disciplines	Professional	– Experience with acute healthcare delivery and medical triage. – Recognition of diseases outside a specific discipline with a broadened differential diagnosis. Allowing understanding of various disciplines and relevant information in communicating with specialists. – Having knowledge of uncommon (infectious) diseases such as TB, Ebola, Measles, Malaria, Kala Azar or Cholera.
2	Holistic perspective	Holistic view of the patient, including determinants of health, beyond the medical issue of the patient only	Professional, cultural	– Taking into account the influences of cultural, societal and socio-economic factors to health (determinants of health) and tailoring treatment to various determinants of health. – Being able to see relations and interconnections between different disciplines. Seeing the patient as a whole, without solely focusing on one organ(system). – Having insight and being aware of healthcare delivery outside the hospital. – Thinking from a patient’s perspective, including their reasons to seek, or not seek help as well as considering their long term goals.
3	Adaptive communication skills	A way of adapting interaction with others with respect to their context or position	Professional, personal, cultural	– Acknowledging the importance of good communication to realise efficient organisation and facilitation of healthcare, e.g. in stressful situations (both team and patient). Learning to communicate goal-oriented and non-offensively. – Assuming an open attitude and learning to listen as well as communicating one’s own boundaries. – Being conscious of various methods of communication and their importance (e.g. body language or being direct). – Learning to communicate with people with different backgrounds (such as superiors in hierarchical systems or patients with a language barrier) and adjust accordingly.
4	Creativity	Thinking of alternatives to get to the desired outcome, improvising, thinking beyond ‘normal’ use of something	Professional, personal	– Learning to improvise when facing challenges, e.g. scarcity of (wound)material or medication. – Thinking out-of-the-box, e.g. employing sport psychologists to provide psychological support to healthcare workers. – Tailoring solutions for individual patients by considering their wishes and needs, such as employing a patient’s social safety net into the patient’s treatment plan.
5	Flexibility	Adapting to changing situations, being compliant and deployable in diverse settings	Professional, personal	– Learning to quickly adapt and familiarise to unknown situations (such as a new team, working culture, country, disease or healthcare system). – Sustaining high work pressure, long workdays and responsibilities as well as learning to delegate tasks or responsibilities. – Learning pragmatic or solution-oriented thinking and working with limited resources. – Being widely/variably employable at different departments and disciplines, as well as assuming various roles such as a pharmacist or manager.
6	Cultural awareness	Being culturally aware and competent	Cultural, professional, personal	– Knowledge in differences in practices, traditions and wishes within other cultures such as interpersonal relations. Respecting and adjusting to differences in backgrounds into account when diagnosing and treating patients. For example, taking on a more paternalistic role as physician in the patient-physician relationship when needed. – Having affinity to and valuing work in diverse settings (places and people). Working flexibly with people from other backgrounds/cultures. – Developing consciousness of the varying ways of communication and expression of feelings and ideas other cultures, such as (body)language or illness experience. Reflecting on these similarities and differences. Overcoming these language and cultural barriers.
7	Self-reliance	Being able to work independently when required	Professional, personal	– Having less need of supervision, with knowledge of one’s own capabilities and limits, such as learning to do rounds or make decisions independently. Being able to take over tasks of supervisors if needed. – Valuing the opportunity of supervision when possible.
8	Clinical competence	Improved clinical skills concerning interpretation of clinical signs, diagnosis and treatment	Professional	– Having multiple years of general work experience with a demanding patient load. Experience in diagnosing based on clinical eye and reasoning, triaging, recognition of acutely ill patient and acting adequately. – Improved surgical and gynaecological skills as well as physical examination and history taking. – Having knowledge and recognition of various stages of diseases (such as eclampsia). Recognising rare diseases/situations (such as breech births). Hands-on experience with a broad spectrum of diseases instead of solely knowing them from literature and their presentations on different skin colours.
9	Cost awareness	Being conscious of the costs of healthcare interventions and treatments	Professional	– Having experience in working with scarcity of (medical) material and consciousness of healthcare costs, such as materials and beds. – Learning to be judicious and cost-effective in use of resources such as material, beds, routine diagnostics as well as unnecessary extension of life. – Learning deliberate practice: switching to suitable care when a treatment is likely to be of little health gain.
10	Public health	Being motivated to strive for health equity within the whole population in a society, including vulnerable groups	Cultural, professional, personal	– Gaining insight into the broader scope of public healthcare and its importance, including communications, sustainability, accessibility, travel medicine, determinants of health, privatisation and infectious diseases. – Acknowledging/emphasising the value of preventive medicine, alongside curative medicine, within a healthcare system. – Affinity with vulnerable groups/migrants and being motivated to provide them access to the healthcare system (such as working in a refugee centre or general practice in a multi-ethnic area). – Learning the importance of and gaining experience in needs assessments in effective developmental aid.
11	Leadership	Working in a team and motivating the team to work toward a common goal	Professional, personal	– Taking care of the team and its members. Conflict-resolving and giving others comfort in situations that are unknown to them. – Experience and confidence in leading or supervising a team: keeping an overview, delegation, prioritization, responsibility, making decisions and being conscious of their consequences. – Being mindful to a team’s long-term goals, motivating and keeping them goal-oriented. Assessing and managing individual skillsets. – Being experienced with educating and training people. Paying attention to the education of others.
12	Open-mindedness	Being open to different perspectives without considering one point of view as absolute	Personal, cultural, professional	– Being able to relate to other perspectives and to think from their point of view (both patient and colleague). Giving attention to what is deemed important by someone else and being disinclined from making value judgements. Being humble and respectful towards opinions, situations, or wishes of others. – Learning the relevance of discrimination/racism and its influence on people. – Having an open attitude toward alternative/complementary medicine and different ways of individual healthcare delivery. – Giving attention and value to the process of the end of life.
13	Organisation of care	Insight into organisation of care and healthcare systems	Professional, cultural	– Being experienced with the logistics, policy-making, cooperation and process improvement within the organisation of healthcare both within as outside of the hospitals (such as the government, public health institutes or NGOs). – Having experience with different aspects of epidemics, including prevention, measures, necessary attitude and organisation of healthcare. Being able to take consulting roles with regard to an epidemic in high-resource settings. – Having experiences with working in situations with limited resources, such as shortage of hospital beds and prioritising healthcare delivery. – Adopting a sustainability-oriented view considering organization of a healthcare system regarding climate change or recycling of materials.
14	Self-development	Process of conscious improvement of personal and professional aspects of life	Personal, professional	– Resilience to stress in both private and professional matters. Being used to high-pressure work environments, such as long working days and substantial responsibility. – Development of self-confidence in various situations, such as acute clinical cases, difficult decisions or uncertainty. – Learning to differentiate between major and minor issues in personal and professional spheres, putting them into perspective. – Having experience with mortality and accepting that death is part of life. Acknowledging the importance and learning to cope with these situations. – Appreciation of services/facilities considered normal in a HRS, such as taking showers with warm drinking water.
15	Teamwork	Collaborating, communicating, working and understanding as a team	Professional, personal, cultural	– Understanding the importance of a safe learning environment as well as shared motivation/team spirit within a team. Adapting communication and work ethic to various kinds of people and hierarchical levels. Learning to work towards a common goal with a conflict resolving, inclusive and structured work ethic. – Being attentive to the mental health of colleagues and learning to cope with stress, uncertainties and frustrations of others. – Improved perspective-taking in communication with other disciplines/roles through experience. – Closing the gap between various disciplines, hospitals and levels of healthcare as well as society and medicine.

## Discussion

This study explored competency development of Ps-GHTM during their work abroad in LRS and the perceived applicability of these competencies in the Netherlands. Ps-GHTM perceive their experience abroad as beneficial for both personal as well as professional development. We describe 15 themes which cover a broad range of competencies: broad medical perspective, holistic perspective, adaptive communication skills, creativity, flexibility, cultural awareness, self-reliance, clinical competence, cost awareness, public health, leadership, open-mindedness, organization of care, self-development, and teamwork. Our findings suggest that a specialisation in GH and work experience abroad, can be of benefit to health care systems in HRS.

Our findings are in line with earlier studies exploring the benefits of experience abroad for medical students and residents. Earlier studies found developments in practical skills, such as physical examination, as well as increased self-confidence, increased affinity with under-served populations, improved management of resources and overcoming language and cultural barriers. The differences between previous work and this study lie mainly in the study population, as Ps-GHTM generally remain in LRS for a longer time. We also describe a broader range of competencies, using data from individual interviews as well as FGDs. Ps-GHTM may develop competencies such as cross-cultural communication, management of epidemics or supervising to a higher level as a result of their specialization as well as the duration of their stay abroad. This may also explain development of competencies such as triage, training/education and awareness of community care.

The competencies we describe are a good fit to the three domains as described by Roy et al. In the following paragraphs, we discuss the relevance of these competencies categorised in these domains. Our findings may also provide indirect evidence of competency development of other specialists in tropical medicine, such as tropical medicine doctors from the United Kingdom. Future investigation of the competencies described here and their implications for healthcare delivery in HRS can further elucidate these benefits.

The professional domain goes beyond the individual professional contribution of the P-GHTM but also relates to health care systems. For example, cost awareness does not only affect choices made by a P-GHTM concerning the use of diagnostic scans or other costly tests. The P-GHTM may also influence the awareness of other health care workers of these costs. Cost-effectiveness is an important factor in reducing health expenditure and a way to reduce costs is raising cost-awareness of medical service providers [42]. Similarly, a broad medical perspective can expand the differential diagnosis and include various diseases seldomly seen in HRS. Knowledge on the global burden of disease is important when striving for health equity and improving health systems, which, in turn, will positively affect the global population [43]. A broad medical perspective can also facilitate interdisciplinary consultation and collaboration that may increase effectivity of care. Experience in acute healthcare and medical triage can enhance the necessary focus and judgement in hectic wards or emergency rooms. Of the three domains, the professional domain is most clearly related to healthcare. Development of the competencies can contribute to overcome the challenges of an increasingly complex and demanding healthcare system.

In the cultural domain participants described that working abroad has enhanced their intercultural competence; improving their communication skills and enabling them to collaborate with people from various cultures, whilst overcoming language barriers. The Netherlands, like many other countries, has a high prevalence of people of foreign descent [44]. Patient-centred care increases patients’ satisfaction of care [45]. Open-mindedness, awareness of patients’ or colleagues’ contextual background and proficiency in cultural awareness helps in providing patient-centred care and may improve teamwork and treatment adherence. Furthermore, communication failures are the most common reason for medical errors [46]. Overcoming cultural and language barriers in healthcare delivery could help reduce such errors. Improving cultural sensitivity and having affinity and working with underserved populations are ways to reduce health inequity [47, 48]. As such, competency development within the cultural domain is increasingly relevant to physicians treating diverse populations.

Health disparities are commonly associated with racism, xenophobia or discrimination which are deeply embedded into every modern healthcare system. This counts for many categorizations, such as ethnicity, religion, gender, and sexual orientation. There is increasing evidence it not only effects the discriminated groups but all groups within. Changing these systems requires doctors that can support legal and political measures and empowerment of affected communities to promote change [9, 10, 12, 48, 47, 48].

Lastly, in the personal domain it is understood that having a global perspective improves understanding of the causes and solutions to local problems [43]. Increased self-confidence and flexibility could prove beneficial in various situations, for example in busy emergency care settings where time is limited or during the COVID-19 pandemic where flexible employment was valued. Lastly, increased stress-resistance is valuable in demanding work environments. Negative emotional states, such as burnouts, not only impede cognitive performance, but also may result in racial bias leading to negative consequences in mindful decision making [49]. Resilience to stress and appreciation of the basic necessities may improve emotional states of Ps-GHTM and aid in mindful decision making. In total, development of competencies within the personal domain improves not only Ps-GHTM, but as an extension their delivery of health care.

The competencies described here often relate to more than one domain, which is to be expected as specific experiences can contribute to development within several domains. For example, cultural awareness not only relates to the cultural domain, but is also an asset within the professional domain. Reflecting the complexity of healthcare delivery, the themes are interrelated and overlapping. The themes also relate to important factors in strengthening health systems worldwide, such as: global and public health, transcendence of professional silo’s, establishing transnational networks and gaining transcultural knowledge [43].

The description of these competencies being developed by working in LRS does not necessarily imply that these competencies cannot be developed in HRS to a similar extent. However, working in LRS may offer a sharp contrast to working in HRS in which these competencies stand out more clearly. The competencies gained in LRS may differ from competencies gained through regular medical training programmes and work experience because of contextual factors. We suggest five contextual factors from our data: assuming a variety of roles and responsibilities, being resource-restricted, having great volumes of hands-on experience, having little to no supervision and being (out of one’s comfort zone) in various settings, traditions and cultures. For example, multiple participants described their time in LRS as a form of ‘pressure cooker’, explaining they gained a significant number of competencies in a relatively short time. On the other hand, many participants noted that not all competencies are directly transferable to their work environment in the Netherlands, such as for example obstetric skills in general practice. Competencies such as communication skills or self-confidence would be easier to apply in both settings. Future research should further investigate the competencies described here and their implications for HRS.

### Strengths and limitations of this study

A scoping review prior to collection of data was done to provide meaning to competencies and to explore a broad spectrum of topics. Examining data from the interviews, validating them in FGDs, and reflecting this back onto our interviews and interview guides has led to comprehensive and rich descriptions of competencies and themes. The interviews were conducted with two researchers throughout 31 of 33 interviews and all FGDs, minimising interviewer bias. The process of coding was done by two researchers, which increased validity of the data. In this study we aimed to inductively create a theory around the experiences on participants. As such, we have continued to concurrently collect and analyse data until no new themes nor codes were found to affirm the adequacy of information power.

A number of limitations remain to this study. The questions asked during the interviews and FGDs could be directed to answer positively as to find supportive data, which could lead to positivity bias. There is considerable heterogeneity within the participants: the amount of time spent in LRS, different countries where they have worked, different roles they served in LRS as well as the Netherlands, two different curricula in which participants have been trained, and two different profiles in the training programme. These were not accounted for, although it may have benefitted the study by enriching the data. Physicians that chose to specialize in GHTM and work abroad for years may have a perspective that over- or underestimates the applicability of their competencies for the Netherlands. Having studied multiple perspectives and discussions between participants combined with a broad spectrum of expertise within the research team reduced this bias.

### Further research

Several new research questions were composed from this study. As this is an initial study, the adopted themes and competencies could be defined more clearly with additional research. Future research is necessary to explore the effects of the developed competencies of Ps-GHTM on the work floor. Further studies could explore these competencies as perceived by other stakeholders that work with Ps-GHTM after their return to a HRS. Another suggestion would be a prospective study following Ps-GHTM to confirm or deepen the themes found in this research. Future studies could explore the relation of the developed competencies to conventional competency-models (e.g., CanMEDS).

Finally, in this research we have studied the competency development and specifically the applicability of these competencies in the Netherlands. Reciprocal benefit is recognized as an important factor in sustainable development of GH and as such, every effort must be made to enhance partnership and sense of ownership for all parties. The needs and priorities of receiving parties as well as the implications for sending parties must be understood to achieve sustainable collaborations.

## Conclusion

This study provides a rich description of the competencies of Ps-GHTM develop in LRS and how these competencies can contribute to healthcare delivery. After their training, Ps-GHTM take their knowledge, skills, and attitudes to contribute to healthcare delivery abroad, commonly in LRS. The competencies they gain could, in turn, contribute to healthcare delivery in the Netherlands. This reciprocal value is important for a sustainable development of GH. Insight in the competencies of Ps-GHTM is essential for all stakeholders, for example in goal-oriented employment of Ps-GHTM. We believe that this study generates a foundation for possible hypothesis generation and, by extension, testing these hypotheses. Acknowledging the importance of GH and globalisation, the mutual benefit of competencies gained abroad and the themes described in this study, Ps-GHTM add to the challenge of striving for health equity worldwide.

## Data Availability

The information letter and letter of informed consent sent to participants, the scoping literature search, as well as the topic guides for the interviews and FGDs are available on request. The data that support the findings of this study are available on reasonable request.

## Declarations

### Reflexivity

HO and LO are medical students from respectively Radboud University and University of Groningen. MB and CT are Ps-GHTM and are well acquainted with the specialisation as well with working abroad. RD is a psychiatrist and researcher with a PhD and MBA in Health Professions Education (HPE). JdZ is a post-doctoral researcher in GH, with a PhD in Medical Sciences and experiences in global health education and qualitative research. MV is a gynaecologist and researcher with experience in qualitative research and a background in international health as well as HPE.

As this is a qualitative study, the data will have been influenced by our backgrounds. LO and HO contributed to this study as medical students with limited experience in research. This may have resulted in a more open and undirected approach to the interviews and analysis. Conversely, this may also have limited depth of the interviews and analysis. We mitigated this by direct supervision during and after the interviews, as well as analysis with experienced researchers.

We applied an interactive and iterative process to this study. This was aided by frequent meetings with the research team where ideas, experience and new insights were discussed. Our study is a product of collaboration by medical students, residents and (medical) supervisors with a broad variation of personal, professional and academic perspectives.

## Abbreviations

FGD: Focus group discussion
GH: Global Health
HRS: High-resource settings
IHE: International health electives
LRS: Low-resource settings
NVTG: Nederlandse Vereniging voor Tropische Geneeskunde en Internationale Gezondheidszorg (the Dutch Association of Tropical Medicine and Global Health)
OIGT: Opleidingsinstituut Internationale Gezondheidszorg en Tropengeneeskunde (the training institute of Ps-GHTM)
P-GHTM: Physician of Global Health and Tropical Medicine

## References

[1] Koplan JP, Bond TC, Merson MH, Reddy KS, Rodriguez MH, Sewankambo NK, Wasserheit JN (2009). Towards a common definition of global health. The Lancet.

[2] Drain PK, Primack A, Hunt DD, Fawzi WW, Holmes KK, Gardner P (2007). Global health in medical education: a call for more training and opportunities. Acad Med.

[3] Liu Y, Zhang Y, Liu Z, Wang J (2015). Gaps in studies of global health education: an empirical literature review. Glob Health Action.

[4] Steenbergen G. Verkenning van het draagvlak voor beleidsformulering. In., 27-07-2018 edn; 2018.

[5] Johnson O, Bailey SL, Willott C, Crocker-Buque T, Jessop V, Birch M, Ward H, Yudkin JS (2012). Global health learning outcomes for medical students in the UK. The Lancet.

[6] Peluso MJ, Van Schalkwyk S, Kellett A, Brewer TF, Clarfield AM, Davies D, Garg B, Greensweig T, Hafler J, Hou J (2017). Reframing undergraduate medical education in global health: Rationale and key principles from the Bellagio Global Health Education Initiative. Med Teach.

[7] Hirsch LA, Martin R. LSHTM and Colonialism: A report on the Colonial History of the London School of Hygiene & Tropical Medicine (1899–c. 1960). 2022.

[8] Bergen, Lv. De oprichting van de ‘Nederlandsche Vereeniging voor Tropische Geneeskunde’: een zaak van nationaal belang. *Studium: Tijdschrift voor Wetenschaps-en Universiteits-geschiedenis| Revue d’Histoire des Sciences et des Universités* 2009, 2(2):92–104. 22586764

[9] Devakumar D, Selvarajah S, Abubakar I, Kim S-S, McKee M, Sabharwal NS, Saini A, Shannon G, White AI, Achiume ET (2022). Racism, xenophobia, discrimination, and the determination of health. The Lancet.

[10] Selvarajah S, Maioli SC, Abi Deivanayagam T, de Morais Sato P, Devakumar D, Kim S-S, Wells JC, Yoseph M, Abubakar I, Paradies Y (2022). Racism, xenophobia, and discrimination: mapping pathways to health outcomes. The Lancet.

[11] Besson EK (2021). Confronting whiteness and decolonising global health institutions. The Lancet.

[12] Shannon G, Morgan R, Zeinali Z, Brady L, Couto MT, Devakumar D, Eder B, Karadag O, Mukherjee M, Peres MFT (2022). Intersectional insights into racism and health: not just a question of identity. The Lancet.

[13] Stein S, Andreotti V, Suša R, Amsler S, Hunt D, Ahenakew C, Jimmy E, Cajkova T, Valley W, Cardoso C (2020). Gesturing towards Decolonial Futures. Nordic J Comp Int Educ (NJCIE).

[14] Eichbaum QG, Adams LV, Evert J, Ho M-J, Semali IA, van Schalkwyk SC (2021). Decolonizing global health education: rethinking institutional partnerships and approaches. Acad Med.

[15] Wigle JM, Akseer N, Carbone S, Barac R, Barwick M, Zlotkin S (2018). Developing a tool to measure the reciprocal benefits that accrue to health professionals involved in global health. BMJ global health.

[16] Sayegh H, Harden C, Khan H, Pai M, Eichbaum QG, Ibingira C, Goba G (2022). Global health education in high-income countries: confronting coloniality and power asymmetry. BMJ Global Health.

[17] Anderson FW, Wansom T (2009). Beyond Medical Tourism: Authentic Engagement in Global Health. AMA J Ethics.

[18] Shah S, Wu T (2008). The medical student global health experience: professionalism and ethical implications. J Med Ethics.

[19] Fu M. Amateurs play doctor for world’s poor. The Daily Beast.The Daily Beast Company; 2017.

[20] Bishop JA, Litch RA (2000). Medical tourism can do harm. BMJ.

[21] Lu PM, Mansour R, Qiu MK, Biraro IA, Rabin TL (2021). Low-and middle-income country host perceptions of short-term experiences in global health: a systematic review. Acad Med.

[22] Crump JA, Sugarman J (2010). Ethics and best practice guidelines for training experiences in Global Health. Am J Trop Med Hyg.

[23] Cherniak W, Latham E, Astle B, Anguyo G, Beaunoir T, Buenaventura J, Decamp M, Diaz K, Eichbaum Q, Hedimbi M (2017). Visiting trainees in global settings: host and Partner Perspectives on Desirable Competencies. Annals of Global Health.

[24] Decamp M, Rodriguez J, Hecht S, Barry M, Sugarman J (2013). An ethics curriculum for short-term global health trainees. Globalization and Health.

[25] Bae C, Naik N, Misak M, Barnes SL, Verceles AC, Papali A, McCurdy MT, Losonczy LI (2020). Assessment of Local Health Worker Attitudes toward International Medical volunteers in low- and middle-income countries: A Global Survey. J Epidemiol Global Health.

[26] Opleiding AIGT. [https://www.oigt.nl/?aigt

[27] Battat R, Seidman G, Chadi N, Chanda MY, Nehme J, Hulme J, Li A, Faridi N, Brewer TF (2010). Global health competencies and approaches in medical education: a literature review. BMC Med Educ.

[28] Law IR, Worley PS, Langham FJ (2013). International medical electives undertaken by australian medical students: current trends and future directions. Med J Aust.

[29] Lu PM, Park EE, Rabin TL, Schwartz JI, Shearer LS, Siegler EL, Peck RN. Impact of Global Health Electives on US Medical Residents: A Systematic Review.Annals of Global Health2018, 84(4). 10.29024/aogh.2379 PMC6748170 30779519

[30] Nordhues HC, Bashir MU, Merry SP, Sawatsky AP (2017). Graduate medical education competencies for international health electives: a qualitative study. Med Teach.

[31] Reardon C, George G, Enigbokan O (2015). The benefits of working abroad for British General Practice trainee doctors: the London deanery out of programme experience in South Africa. BMC Med Educ.

[32] Roy A, Newman A, Ellenberger T, Pyman A (2018). Outcomes of international student mobility programs: a systematic review and agenda for future research. Stud High Educ.

[33] Thompson MJ, Huntington MK, Hunt DD, Pinsky LE, Brodie JJ (2003). Educational effects of international health electives on US and canadian medical students and residents: a literature review. Acad Med.

[34] van den Hombergh P, de Wit NJ, van Balen FA (2009). Experience as a doctor in the developing world: does it benefit the clinical and organisational performance in general practice?. BMC Fam Pract.

[35] Ghaljaie F, Naderifar M, Goli H. Snowball Sampling: a Purposeful Method of Sampling in qualitative research. Strides in Development of Medical Education. 2017;14(3):–.

[36] Tavakol M, Sandars J (2014). Quantitative and qualitative methods in medical education research: AMEE Guide No 90: part II. Med Teach.

[37] Sullivan GM, Sargeant J (2011). Qualities of qualitative research: part I. J Grad Med Educ.

[38] Frank JR, Snell L, Sherbino J (2015). CanMEDS 2015 Physician Competency Framework.

[39] Hsieh HF, Shannon SE (2005). Three approaches to qualitative content analysis. Qual Health Res.

[40] Tavakol M, Sandars J (2014). Quantitative and qualitative methods in medical education research: AMEE Guide No 90: part I. Med Teach.

[41] Baarda B (2018). Basisboek kwalitatief onderzoek: handleiding voor het opzetten en uitvoeren van kwalitatief onderzoek, vol. Vierde druk.

[42] Stammen LA, Stalmeijer RE, Paternotte E, Oudkerk Pool A, Driessen EW, Scheele F, Stassen LPS (2015). Training Physicians to provide High-Value, cost-conscious care. JAMA.

[43] Frenk J, Chen L, Bhutta ZA, Cohen J, Crisp N, Evans T, Fineberg H, Garcia P, Ke Y, Kelley P (2010). Health professionals for a new century: transforming education to strengthen health systems in an interdependent world. The Lancet.

[44] Statistics CBf. Bevolking; geslacht, leeftijd, generatie en migratieachtergrond [Data file]. In.: Centraal Bureau voor de Statistiek; 2020.

[45] Epstein RM, Street RL (2011). The values and value of patient-centered care. The Annals of Family Medicine.

[46] Leonard M, Graham S, Bonacum D (2004). The human factor: the critical importance of effective teamwork and communication in providing safe care. BMJ Qual Saf.

[47] Vanderbilt AA, Baugh RF, Hogue PA, Brennan JA, Ali II (2016). Curricular integration of social medicine: a prospective for medical educators. Med Educ Online.

[48] Abubakar I, Gram L, Lasoye S, Achiume ET, Becares L, Bola GK, Dhairyawan R, Lasco G, McKee M, Paradies Y (2022). Confronting the consequences of racism, xenophobia, and discrimination on health and health-care systems. The Lancet.

[49] Dyrbye L, Herrin J, West CP, Wittlin NM, Dovidio JF, Hardeman R, Burke SE, Phelan S, Onyeador IN, Cunningham B (2019). Association of racial Bias with Burnout among Resident Physicians. JAMA Netw Open.

