# Publisher Correction: Applicability of working abroad for physicians with a specialization in global health and tropical medicine

**DOI:** 10.1186/s12992-023-00932-x

**Published:** 2023-05-23

**Authors:** Hasan Özcan, Loes Overeem, Maria Bakker, Caroline Telkamp, Robbert Duvivier, Janine de Zeeuw, Marco Versluis

**Affiliations:** 1 Radboud University, Nijmegen, The Netherlands; 2 University of Groningen, Groningen, The Netherlands; 3 Department of Paediatrics, Maasstad Ziekenhuis, Rotterdam, The Netherlands; 4 Department of Obstetrics and Gynaecology, St. Walburg’s Hospital, Nyangao, Tanzania; 5 Department of Health Sciences, University Medical Center Groningen, Groningen, The Netherlands; 6 Department of Health Sciences, Global Health Unit, University Medical Center Groningen, Groningen, The Netherlands; 7 Department of Obstetrics and Gynaecology, University Medical Center Groningen, Hanzeplein 1, Groningen, 9713 GZ the Netherlands

Globalization and Health (2023) 19:28

10.1186/s12992-023-00929-6

Following publication of the article, it came to the publisher’s attention that the article had been produced with an error in the author list: the author Marco Versluis had been positioned in first place, while they should be positioned last to reflect their role as supervisor. The author list has since been corrected in the published article and the corrected author list may be seen in this erratum. The publisher thanks you for reading and apologizes for any inconvenience caused.

